# Prognostic Impact of Vaccination, Comorbidity, and Inflammatory Biomarkers on Clinical Outcome in Hospitalized Patients with COVID-19

**DOI:** 10.3390/biomedicines13081995

**Published:** 2025-08-16

**Authors:** Sandra Bižić-Radulović, Tijana Subotički, Olivera Mitrović Ajtić, Teodora Dragojević, Emilija Živković, Sanja Miljatović, Dalibor Petrović, Dejana Stanisavljević, Snežana Jovanović, Milanko Šekler, Dejan Vidanović, Bojana Beleslin Čokić, Vladan P. Čokić

**Affiliations:** 1 Clinic of Hematology, University Clinical Centre of Serbia, 11000 Belgrade, Serbia; bizics@yahoo.com; 2 Institute for Medical Research, University of Belgrade, 11000 Belgrade, Serbia; tijana@imi.bg.ac.rs (T.S.); oliveram@imi.bg.ac.rs (O.M.A.); teodora.dragojevic@imi.bg.ac.rs (T.D.); emilija.zivkovic@imi.bg.ac.rs (E.Ž.); 3 Clinic for Infectious and Tropical Diseases, University Clinical Centre of Serbia, 11000 Belgrade, Serbia; sanjadrmanic@yahoo.com; 4 Faculty of Transport and Traffic Engineering, University of Belgrade, 11000 Belgrade, Serbia; dalibor73@gmail.com; 5 Institute for Medical Statistics and Informatics, Faculty of Medicine, University of Belgrade, 11000 Belgrade, Serbia; dejana.stanisavljevic@med.bg.ac.rs; 6 Department of Microbiology, University Clinical Centre of Serbia, 11000 Belgrade, Serbia; drsnezana.jovanovic@gmail.com; 7 Veterinary Specialized Institute “Kraljevo”, 36000 Kraljevo, Serbia; sekler@vsikv.com (M.Š.); vidanovic@vsikv.com (D.V.); 8 Institute of Virology, Vaccines and Sera “Torlak”, 11000 Belgrade, Serbia; bbeleslin@torlak.rs

**Keywords:** COVID-19, inflammation, SARS-CoV-2 variants, comorbidities, vaccination, sex difference

## Abstract

**Background/Objectives**: The coronavirus disease 2019 (COVID-19) has more severe symptoms and increased mortality among men than women. To address the prognostic impact of vaccination, comorbidities, and inflammatory biomarkers on classified clinical outcomes in hospitalized COVID-19 patients, we compared common and sex differences. **Methods**: Besides laboratory and clinical parameters at hospital admission, we performed a common and sex-based comparative analysis for the clinical outcomes, RT-qPCR analyses, and measured severe acute respiratory syndrome coronavirus (SARS-CoV-2)-specific IgM and IgG antibody levels of 702 COVID-19 patients in a single centre from June 2020 to April 2022. **Results:** Pro-inflammatory biomarkers (C-reactive protein (CRP), interleukin-6 (IL-6), fibrinogen, lactate dehydrogenase (LDH), D-dimer, ferritin), and liver enzymes (AST, ALT, GGT) were significantly more increased in COVID-19 male patients and generally elevated with the severity of clinical outcome, regardless of the SARS-CoV-2 variant. Cycle threshold (Ct) values of RT-qPCR testing were in negative correlation with IL-6 in COVID-19 male patients, indicating that higher viral load largely increased IL-6 levels in parallel with the severity of clinical outcome and regardless of vaccination. IgG levels were higher in early post-COVID-19 male patients. Comorbidities were more frequent in COVID-19 female patients and generally more common in the severe clinical outcomes. Vaccination was negatively correlated with the severity of clinical outcome, liver enzymes, LDH, and inflammatory parameters in hospitalized COVID-19 patients, while the risk of pneumonia was reduced. Vaccination reduced the need for corticosteroid and anti-inflammatory therapies, but increased the need for antiviral drug treatment. **Conclusions**: In addition to confirming inflammatory biomarkers and the importance of anti-inflammatory therapy in vaccinated patients, this study showed that vaccination reduces, but does not prevent, mortality in patients with COVID-19.

## 1. Introduction

Immunopathology of coronavirus disease 2019 (COVID-19) is defined by the involvement of immune cells initiating a local and systemic inflammatory response, with risk factors such as age, male sex, and comorbidities [1]. Meta-analysis on 229 studies involving over 10 million patients shows that men have a higher risk for COVID-19 infection, hospitalization, severe outcome, intensive care, and death compared to women [2]. In the pre-vaccine period, COVID-19 mortality was higher for males, but it was partly attenuated in the post-vaccine period [3]. In addition to increased mortality, the risk of thrombosis is also elevated in men, which is most evident in younger age groups infected by severe acute respiratory syndrome coronavirus (SARS-CoV-2) [4]. It has been demonstrated that testosterone and free testosterone levels are reduced in hospitalized COVID-19 male patients but recover after a couple of months [5]. Moreover, deceased males have lower testosterone and higher estradiol than survivors, while testosterone is negatively associated with severe COVID-19 and in-hospital mortality [6]. Regarding inflammation biomarkers, testosterone is inversely associated with interleukin-6 (IL-6) and C-reactive protein (CRP) in severe COVID-19, while testosterone and estradiol are similar in women with and without severe COVID-19 [7]. Consistent with this association, men with severe COVID-19 disease have significantly higher levels of pro-inflammatory cytokines (IL-6, IL-8, monocyte chemoattractant protein-1 (MCP-1)), while women have significantly higher levels of the anti-inflammatory cytokine IL-10 [8].

Sex differences are also present in comorbidities such as liver disease, dementia, metastatic solid tumor, heart failure, and others that show a higher level of mortality risk in women compared to men [9,10]. Related to post-COVID syndrome, COVID-19 sequelae in the psychiatric/mood, musculoskeletal, and respiratory categories are more probable among women, whereas renal sequelae are more expected among men [11]. Post-COVID-19 women are more affected by anxiety/depression in their usual activities [12]. Considering the immune response to SARS-CoV-2 infection, immunoglobulin G (IgG) antibodies rise sharply and peak faster in female patients, while they increase gradually and peak later in male patients [13].

Clinical outcome is very negative for men, which is improved by COVID-19 vaccination. This study aims to observe sex differences in the prognostic impact of vaccination, comorbidities, and inflammatory biomarkers on classified clinical outcomes in patients with COVID-19 infection at hospital admission. Sex difference is evaluated according to laboratory and clinical data, therapy, comorbidities, vaccination, and severity of clinical outcome in 702 patients hospitalized for COVID-19, expanded by reverse transcription–quantitative polymerase chain reaction (RT-qPCR) analyses and antibody testing of SARS-CoV-2 variants. The frequency of comorbidities and intensity of immune response demonstrate the opposite results in COVID-19 female and male patients.

## 2. Materials and Methods

### 2.1. Sample Collection

Peripheral blood samples from 702 patients with COVID-19, confirmed by positive RT-qPCR and/or antibody testing, were collected at the Clinic for Infectious and Tropical Diseases, University Clinical Centre of Serbia, Belgrade, Serbia. A flowchart with inclusion and exclusion criteria for hospitalization of patients with COVID-19 is provided in Supplemental Scheme S1. The average age of male patients with COVID-19 was 60.3 ± 17.2 (max 94, min 18), while the median was 63 (interquartile range (IQR): 47–74). The average age of female patients with COVID-19 was 62.8 ± 17.2 (max 99, min 20), while the median was 67 (IQR: 50–75). Information on patients’ clinical and laboratory data, achieved in the first week of COVID-19 infection, was extracted from their medical records, including vaccination status (categorized as 0—not vaccinated; 1—one dose; 2—two doses of vaccine; and 3—booster). The World Health Organization (WHO) ranked the severity of the clinical outcomes (ranked as 1—mild; 2—moderate; 3—severe; 4—critical; and 5—deceased) and comorbidities at diagnosis (present or absent, and specific). Normal values of laboratory data were presented in our previous COVID-19 study [5]. This study was conducted in accordance with the Declaration of Helsinki and approved by the Ethics Committee of the University Clinical Centre of Serbia (protocol code 570/12) and the Institute for Medical Research (131/2020). This study included 702 hospitalized patients (275 females and 427 males) with COVID-19 examined between June 2020 and April 2022. The patients were diagnosed according to the Clinical Management of COVID-19 guidelines by the WHO. All patients signed an informed consent approved by the institutional review board. The male patients’ sample volume was greater compared to the female patients’, showing that most hospitalized patients with COVID-19 were males. We categorized 702 hospitalized patients with COVID-19 in 5 groups according to therapy and clinical outcome: (1) antiviral—mild form; (2) antiviral and corticosteroids—moderate form; (3) corticosteroids, IL-6 inhibitor (Tocilizumab), and oxygen—severe form; (4) intensive care unit (ICU), corticosteroids, IL-6 inhibitor, ventilation—critical form; (5) ICU, corticosteroids, IL-6 inhibitor, ventilation—deceased.

### 2.2. RT-PCR Testing

Nasopharyngeal and oropharyngeal swabs were collected from patients suspected to have COVID-19 infections, and specimens were stored together in a collection tube. The BGI testing was performed as previously described and in accordance with the manufacturer’s instructions [14]. Briefly, viral RNA was isolated using an MGIEasy Nucleic Acid DNA/RNA Extraction Kit (MGI Tech Co., Ltd., Shenzhen, China) and MGISP-960 automatic extraction approach (MGI Tech Co., Ltd.). The RT-PCR amplification was conducted using the fluorescent quantitative detection system (LineGene 9600 Plus, Hangzhou Bioer Technology Co., Ltd., Hangzhou, China). A sample was confirmed positive for COVID-19 when the cycle threshold (Ct) value of the target ORF1ab region was ≤ 35.

### 2.3. IgM/IgG ELISA Assay

Peripheral blood samples were obtained from COVID-19 patients. Blood samples were collected with ethylenediaminetetraacetic acid (EDTA), and plasma was separated by centrifugation at 2000 rpm for 15 min. The samples were stored at −80 °C until analysis. Immunoglobulin M (IgM) levels were measured using a SARS-CoV-2 Nucleocapsid Protein IgM ELISA Kit (Elabsciences Biotechnology Inc., Houston, TX, USA, Cat. No. E-EL-E601), according to the manufacturer’s instructions. IgG levels were measured using a SARS-CoV-2 Nucleocapsid Protein IgG ELISA Kit (Elabsciences Biotechnology Inc., Houston, TX, USA, Cat. No. E-EL-E600), according to the manufacturer’s instructions. The level of IgM in plasma was measured at the time of hospital admission, while the levels of IgG were measured 2.5 and 5 months after hospital admission. In both analyses, each result was analyzed independently and determined according to a given cutoff value (the cutoff value for IgM was 0.2, and for IgG 0.22). Measurements were performed on an ELISA Multiscan Plus plate reader (Labsystems, Vantaa, Finland).

### 2.4. Whole-Genome Sequencing of SARS-CoV-2 Isoforms

Extraction of viral RNA, complementary DNA (cDNA) synthesis, multiplex PCR, nanopore library preparation, and reference-based assembly were performed as already described [15]. Briefly, viral RNA was extracted from 16 samples using a commercial kit, BIOEXTRACT^®^ SUPERBALL^®^, according to the manufacturer’s instructions (Biosellal, Dardilly, France) using Kingfisher Flex device (Thermo Fisher Scientific, Vantaa, Finland). Library preparation and whole-genome sequencing was performed following Eco PCR tiling of SARS-CoV-2 virus with native barcoding (Oxford Nanopore Technologies, Oxford, UK, Version: TCE_9122_v109_revC_10Feb2020) on a Minion device (Oxford Nanopore Technologies). Bioinformatics analysis was performed using ARTIC nCoV bioinformatics standard operating procedure v.1.1.0 [16]. Genome sequences were deposited in the GISAID database (the Global Initiative on Sharing all Individual Data, available at https://www.gisaid.org under the accession numbers from EPI_ISL_18909196 to EPI_ISL_18909211, accessed on 18 February 2024) and GenBank database (https://www.ncbi.nlm.nih.gov/genbank/ under the accession numbers from PP346032 to PP346047, accessed on 18 February 2024).

### 2.5. Statistical Analysis

The normality of data distribution was examined by the Shapiro–Wilk and Kolmogorov–Smirnov tests, and data from figures and tables generally do not have normal distribution, except IgG results for females. The results are expressed as median with IQR and mean ± standard error of mean (SEM). Differences between groups were analyzed using Student’s *t*-test. When the distribution was not normal, the Mann–Whitney test was used for intergroup comparisons. The correlations between numerical variables were assessed by Spearman’s correlation coefficients. The False Discovery Rate (FDR) was used in multiple hypothesis testing to control the expected proportion of incorrectly rejected null hypotheses. The method of the two-stage linear step-up procedure of Benjamini, Krieger, and Yekutieli was applied for desired FDR (Q = 5%). Multivariable logistic regression was used, with the severity of clinical outcome as the dependent variable (mild/moderate vs. severe/critical/deceased). Results were expressed as odds ratios (ORs) at 95% confidence intervals (CI). All tests were two-tailed. *p* < 0.05 was considered statistically significant. The IBM SPSS statistics version 25 (Chicago, IL, USA) package and GraphPad Prism version 8.0.0 for Windows (GraphPad Software Inc., San Diego, CA, USA) were used for these analyses.

## 3. Results

### 3.1. Sex Difference in Inflammation Biomarkers of COVID-19 Patients at Hospital Admission

**Figure 1:**
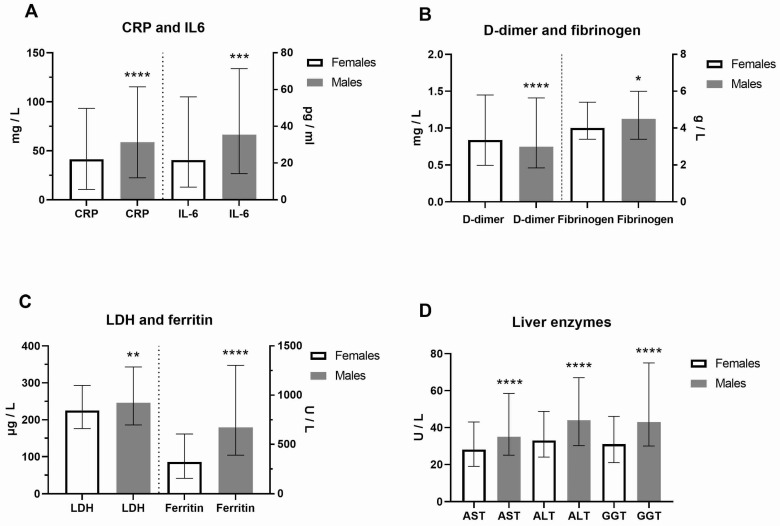
Sex difference in inflammation markers of patients with COVID-19 on hospital admission. Inflammatory biomarkers are examined in females (F) and males (M, n = F/M) through (**A**) C-reactive protein (CRP, n = 275/426 patients) and interleukin-6 (IL-6, n = 199/340) levels; (**B**) D-dimer (n = 273/425) and fibrinogen (n = 274/417) levels; (**C**) Lactate dehydrogenase (LDH, n = 271/424) and ferritin (n = 196/308) levels; (**D**) Liver enzymes aspartate aminotransferase (AST, n = 271/425), alanine transaminase (ALT, n = 272/424), and gamma-glutamyltransferase (GGT, n = 255/396) levels. Values are median with interquartile range. **** *p* < 0.0001, *** *p* < 0.001, ** *p* < 0.01, * *p* < 0.05 vs. COVID-19 paired females determined by Mann–Whitney test.

Clinical and laboratory data of COVID-19 female and male patients at hospital admission were presented in Figure 1 and Supplemental Table S1. In contrast to platelets, the neutrophil percentage was higher in males. As a result of increased activity of coagulation factors, prothrombin time (PT) was decreased, while activated partial thromboplastin time (aPTT) was prolonged in males (*p* < 0.0001). In addition, urea and creatinine were higher in males, as well as creatine kinase (CK), reflecting the muscle aches typical for COVID-19. CRP (Mann–Whitney U = 49892, *p* = 0.0009) and IL-6 (Mann–Whitney U = 27168, *p* = 0.0001) were also significantly increased in males (Figure 1A). Increased fibrinogen levels (Mann–Whitney U = 51098, *p* = 0.0187), typical in inflammatory states, were generally increased, but more in COVID-19 male patients, and linked to pathological thrombosis (Figure 1B). Fatigue and joint pain are common symptoms of COVID-19 related to double-increased ferritin levels in males (Figure 1C). As a marker of inflammation, lactate dehydrogenase (LDH) levels were increased dominantly in males (Mann–Whitney U = 49998, *p* = 0.0039, Figure 1C). The liver enzymes alanine transaminase (ALT), aspartate aminotransferase (AST), and gamma-glutamyl transferase (GGT, *p* < 0.0001, Figure 1D) were also significantly increased in males. However, we did not find a significant sex difference in clinical outcome (*p* = 0.2402). Comorbidities were more increased in COVID-19 female patients (Supplemental Table S1). The most frequent comorbidities in females were cardiovascular disorders (57.8%, of which hypertension was 92.5%), diabetes mellitus (42.2%), obesity (22.2%), endocrine disorders (16.4%), cancers (13.8%) and pulmonary disorders (11.3%). The most frequent comorbidities in males were also cardiovascular disorders (51.5%, hypertension 93.6%), diabetes mellitus (16.6%), obesity (18.7%), urogenital disorders (10.1%), and cancers (9.8%) (Supplemental Table S2).

### 3.2. Sex Difference in Inflammation Biomarkers of COVID-19 Patients in Accordance with Clinical Outcome

**Figure 2:**
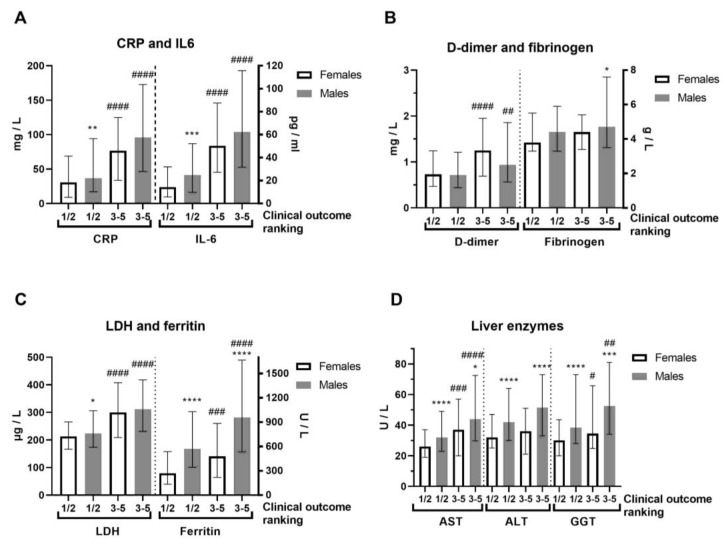
Sex difference in inflammation markers of patients with COVID-19, on hospital admission, according to severity of clinical outcome. The clinical outcomes were ranked as 1—mild; 2—moderate; 3—severe; 4—critical; and 5—deceased, while we gathered them in two forms: 1/2 (mild, n = 192–196/286–293) and 3–5 (severe, n = 78–79/131–134) in females/males (n = F/M). (**A**) C-reactive protein (CRP) and interleukin-6 (IL-6, n = 140/222 form 1/2 and 59/118 form 3–5) levels; (**B**) D-dimer and fibrinogen levels; (**C**) Lactate dehydrogenase (LDH) and ferritin (n = 137/202 form 1/2 and 59/106 form 3–5) levels; (**D**) Liver enzymes aspartate aminotransferase (AST), alanine transaminase (ALT) and gamma-glutamyltransferase (GGT, n = 177/290 form 1/2 and 78/106 form 3–5) levels. Values are median with interquartile range. **** *p* < 0.0001, *** *p* < 0.001, ** *p* < 0.01, * *p* < 0.05 vs. COVID-19 paired females. #### *p* < 0.0001, ### *p* < 0.001, ## *p* < 0.01, # *p* < 0.05 vs. paired same sex form ½, determined by Mann–Whitney test.

**Figure 3:**
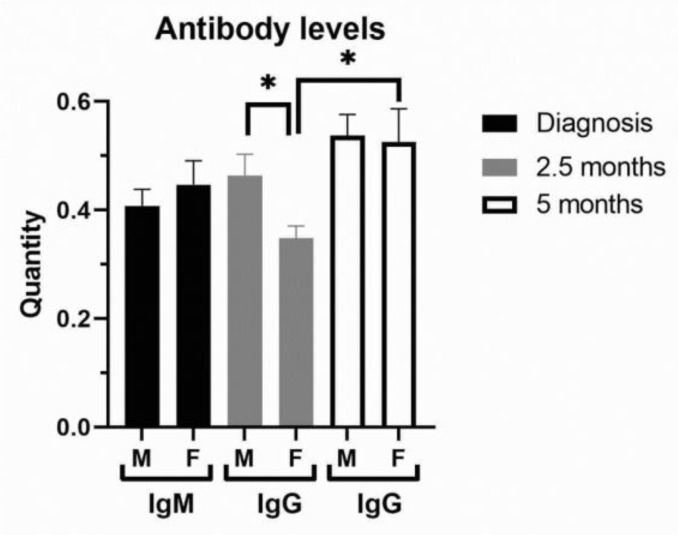
Antibody levels in peripheral blood of COVID-19 and post-COVID-19 patients. The levels of IgM were examined in COVID-19 female (F, n = 26) and male (M, n = 41) patients at hospital admission, while IgG levels were examined in post-COVID-19 patients after 2.5 and 5 (n = 13 for F, n = 26 for M) months of hospital admission. Values are mean ± SEM. * *p* < 0.05 vs. COVID-19 female patients after 2.5 months determined by Mann–Whitney test (M/F) and paired *t* test (F/F).

In terms of severity of clinical outcome, we analyzed inflammation biomarkers of COVID-19 patients (Figure 2, Supplemental Table S3). Comorbidities were generally more frequent in COVID-19 female patients (Supplemental Table S3). COVID-19 female patients with a mild (1–2) outcome had 3-fold decreased IL-6 compared to female patients with a severe clinical outcome (3–5) and 2-fold lower compared to COVID-19 male patients with a mild outcome (Figure 2A). Severe clinical outcome (3–5) is significantly higher in less-vaccinated male patients, compared to patients with a mild clinical outcome (1–2, Supplemental Table S3). Fibrinogen was more increased in COVID-19 male patients with severe clinical outcome (Figure 2B). Creatinine and CK were also increased in the severe clinical outcome and significantly more in COVID-19 male patients (Supplemental Table S3). CRP (Mann–Whitney U = 24655, *p* = 0.008), IL-6 (Mann–Whitney U = 12010, *p* = 0.0003), and ferritin (Mann–Whitney U = 7585, *p* < 0.0001) were largely increased in severe COVID-19 male patients (Figure 2A,C). Moreover, liver enzymes AST, ALT, and GGT were significantly more increased in COVID-19 male patients (Figure 2D). Antibody testing demonstrated a similar sex response of IgM to SARS-CoV-2 infection (Figure 3). It has been observed that the sex difference in IgG response after 2.5 months of hospital admission favors post-COVID-19 male patients (Mann–Whitney U = 87, *p* = 0.0135). However, the IgG response lost sex dependence after 5 months of hospital admission, and IgG levels were further increased in post-COVID-19 female patients (paired *t* test, *p* = 0.0282, Figure 3).

### 3.3. Laboratory Data of COVID-19 Patients in Correlation with Clinical Outcome, PCR Testing, and Vaccine Status

Clinical outcome was generally in positive correlation with IL-6 and LDH, while in negative correlation with Ct values of RT-qPCR testing and application and quantity of vaccines in COVID-19 male patients (Table 1). Regarding COVID-19 male patients, white blood cells (WBCs) were in positive correlation with Ct values of RT-qPCR testing and application and quantity of vaccines (Table 1). IL-6 was in negative correlation with Ct values of RT-qPCR testing (Table 1). Creatinine, D-dimer, and CRP were in positive correlation with clinical outcome of male COVID-19 and IL-6. CK, AST, ALT, and GGT were in negative correlation with application and quantity of vaccines. Ferritin, fibrinogen, and LDH were in positive correlation with IL-6 and clinical outcome of male COVID-19, while ferritin and LDH were in negative correlation with application and quantity of vaccines (Table 1). Creatinine, D-dimer, and CRP were positively correlated with clinical outcome of female COVID-19 and IL-6 (Table 2). Moreover, AST and GGT were in positive correlation with IL-6 and clinical outcome, while AST and ALT were in negative correlation with application and quantity of vaccines (Table 2). The correlation r values are generally below 0.3, while those above 0.3 (13.8%) are linked to well-known inflammatory markers (CRP, LDH, and IL-6). In contrast, *p* values of correlation are generally high: 33.9% of *p* < 0.0001, 15.5% of *p* < 0.001, and 21.7% of *p* < 0.01. In contrast to Ct values, application of FDR generally reduced the q values in the correlation of laboratory parameters with the severity of clinical outcome and IL-6, but had a mixed effect on vaccine status (Supplemental Table S4). The severity of clinical outcome was significantly more pronounced in unvaccinated than in vaccinated COVID-19 patients (Mann–Whitney U = 54058, *p* = 0.0062).

### 3.4. Comorbidities and Therapy of COVID-19 Patients in Correlation with Clinical Outcome

Comorbidities were generally in positive correlation with the clinical outcome, as well as in female COVID-19 patients, while in male COVID-19 patients were positively correlated with IL-6 (Supplemental Table S5). Comorbidities related to the cardiovascular system and urinary–genital system were in significant positive correlation only with the clinical outcome of COVID-19 female patients. Diabetes mellitus was in significant positive correlation with the IL-6 of COVID-19 female patients. Comorbidities related to blood and blood-forming organs were generally in positive correlation with the clinical outcome and IL-6 of COVID-19 patients. Comorbidities related to pulmonary and psychiatric diseases were generally and individually in positive correlation with the clinical outcome of COVID-19 female and male patients (Supplemental Table S5). Comparing vaccinated and unvaccinated hospitalized patients with COVID-19, we showed that 205/292, 130/113 and 48/93 of them received corticosteroid, antiviral, and anti-inflammatory therapy, respectively. Vaccination was in negative correlation with corticosteroid (Spearman r = −0.08, 95% CI = −0.157–0.003, *p* = 0.036) and anti-inflammatory (Spearman r = −0.098, 95% CI = −0.174–0.02, *p* = 0.011) therapy, but in positive correlation with antiviral therapy (Spearman r = 0.156, 95% CI = 0.079–0.231, *p* < 0.0001). Moreover, the number of vaccine doses was in an even stronger negative correlation with corticosteroid (Spearman r = −0.282, 95% CI = −0.368–0.191, *p* < 0.0001) and anti-inflammatory (Spearman r = −0.173, 95% CI = −0.264–0.078, *p* = 0.0003) therapy, but in positive correlation with antiviral therapy (Spearman r = 0.225, 95% CI = 0.131–0.314, *p* < 0.0001). In summary, vaccination reduced therapy by corticosteroids and anti-inflammatory drugs, but increased treatment by antiviral drugs.

### 3.5. Logistic Regression Models with Clinical Outcome and Laboratory Parameters as Dependent Variables of COVID-19 Patients

By multivariate logistic regression analysis, we examined relationships between the severity of clinical outcome and influence factors of the COVID-19 patients at hospital admission (Table 3). Age was generally significantly associated with the severity of clinical outcome (*p* = 0.004) as well as in COVID-19 female patients (*p* = 0.002). The multivariable logistic regression demonstrated that urea, CRP, and LDH showed significant positive relationships (*p* < 0.001) with the severity of clinical outcome both generally and in COVID-19 male patients, as well as LDH in COVID-19 female patients (Table 3). Vaccination demonstrated negative relationship with the severity of clinical outcome both generally (*p* = 0.003) and in COVID-19 male patients (*p* = 0.043), as well as lymphocytes in COVID-19 female patients (*p* = 0.001, Table 3). As inclusion criteria (Supplemental Scheme S1), a CT severity score ≥8 is commonly used as an indication of clinically relevant COVID-19 pneumonia, verified in 106 unvaccinated and 71 vaccinated hospitalized COVID-19 patients examined (Supplemental Table S6). The CT severity score had a median of 12 (IQR: 9–15) for unvaccinated patients, while the median was 12 (IQR: 10–15) for vaccinated patients. The CT severity score was in significant and positive correlation with the severity of clinical outcome (Spearman r = 0.3806, 95% CI = 0.213–0.526, *p* < 0.0001) for unvaccinated COVID-19 patients, but not for vaccinated COVID-19 patients (Spearman r = 0.2026, 95% CI = −0.02–0.406, *p* = 0.0662). Therefore, vaccination reduced the risk of pneumonia in hospitalized patients with COVID-19.

### 3.6. Sex Difference in Laboratory and Clinical Parameters of COVID-19 Patients in Accordance with SARS-CoV-2 Variants

**Figure 4:**
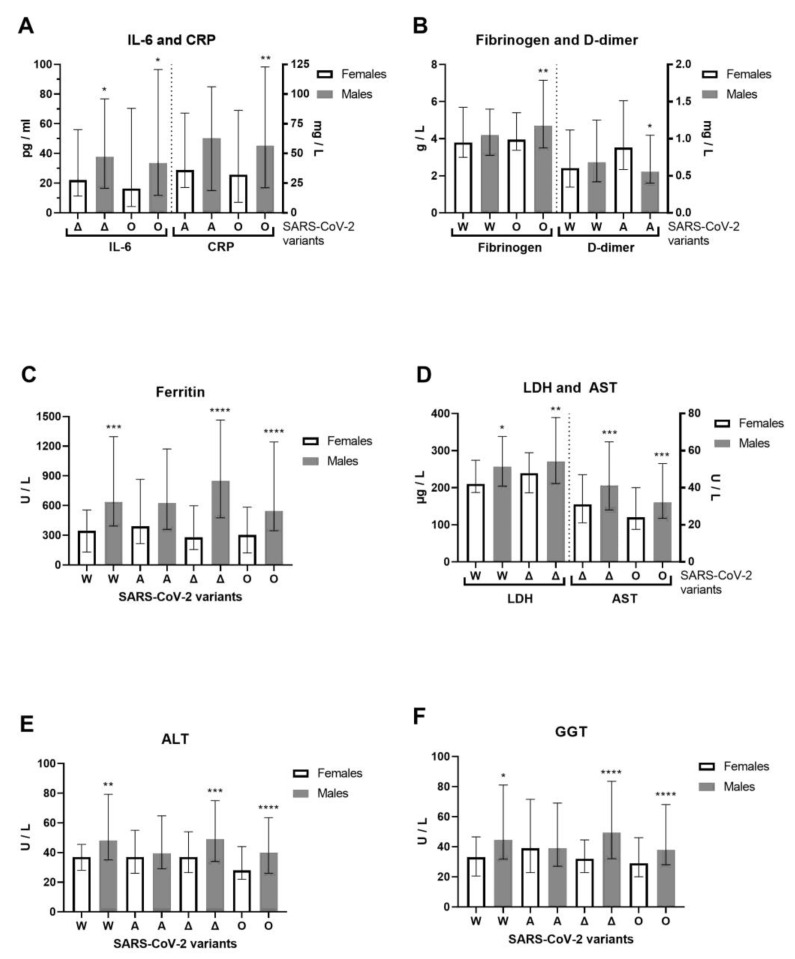
Sex difference in inflammation markers of patients with COVID-19, on hospital admission, in accordance with SARS-CoV-2 variants. The following inflammation markers are examined in accordance to SARS-CoV-2 variants in females/males (n = F/M): Wuhan-1 (W, n = 21–30/47–72), alpha (A, n = 37–41/63–78), delta (Δ, n = 64–78/107–131), and omicron (O, n = 71–126/91–145): (**A**) C-reactive protein and interleukin-6 and C-reactive protein levels; (**B**) fibrinogen and D-dimer levels; (**C**) ferritin; (**D**) lactate dehydrogenase (LDH) and aspartate aminotransferase (AST) levels; (**E**) alanine transaminase (ALT) and (**F**) gamma-glutamyltransferase (GGT) levels. Values are median with interquartile range. **** *p* < 0.0001, *** *p* < 0.001, ** *p* < 0.01, * *p* < 0.05 vs. COVID-19 paired females determined by Mann–Whitney test.

We examined hospitalized COVID-19 patients between June 2020 and April 2022 that allow us to cover four previously circulating SARS-CoV-2 variants of concern (VOCs) [16]: Wuhan-1 (A.1–A.6), alpha (lineage B.1.1.7), delta (lineage B.1.617.2) and omicron (lineage B.1.1.529) in the Phylogenetic Assignment of Named Global Outbreak (PANGO) Lineages nomenclature system, confirmed by whole-genome sequencing for 11 alpha and 5 delta SARS-CoV-2 variants. For the alpha variant, 9 B.1.1.7 (alpha variant) and 2 B.1.1.7.7 sublineages (genetic descendant of the alpha variant) were detected. The alpha variant was one of the first major VOCs, while B.1.1.7.7 was not considered a VOC. The median clinical outcome was 2.5 (IQR: 2–3) for patients with the B.1.1.7 variant and 3 for patients with the B.1.1.7.7 sublineage in generally unvaccinated patients. For the delta variant, three B.1.617.2.122 sublineages and two B.1.617.2.46.6 sub-sublineages were detected. Both are not considered VOCs. The clinical outcome was moderate (median 2, IQR: 2–2) for patients with the B.1.617.2.122 sublineage where two of the three patients were vaccinated. The clinical outcome was critical (median 4, IQR: 3–5) for patients with the B.1.617.2.46.6 sub-sublineage. The unvaccinated patient died, while the vaccinated patient with B.1.617.2.46.6 had a severe clinical outcome. During the Wuhan-1 SARS-CoV-2 variant of 2020, we observed 102 patients with COVID-19 (72 males and 30 females, with an average age of 53.6 ± 18 and 53.6 ± 15.8, respectively). During the alpha SARS-CoV-2 variant (first half of 2021), we observed 119 patients with COVID-19 (78 males and 41 females, with an average age of 61 ± 14.9 and 63.6 ± 13.7, respectively). During the delta SARS-CoV-2 variant (second half of 2021) we observed 209 patients with COVID-19 (131 males and 78 females, with an average age of 54 ± 15.9 and 55.8 ± 17.4, respectively). During the omicron SARS-CoV-2 variant (first half of 2022), we observed 272 patients with COVID-19 (146 males and 126 females, with an average age of 68.9 ± 15.3 and 69 ± 15.9, respectively). Younger patients were more infected with COVID-19 during the first wave of the Wuhan-1 variant, when vaccines were unavailable, and during the delta variant wave, when vaccines were available. The severity of clinical outcome was significantly more pronounced in male than in female patients with the delta SARS-CoV-2 variant (Mann–Whitney U = 4395, *p* = 0.0395). Of 702 patients with COVID-19, 298 were infected and hospitalized despite vaccination. Previously, 17 patients had received one dose of the vaccine, 128 patients had received two doses of the vaccine, and 153 patients had received three doses of the vaccine. The AstraZeneca vaccine was previously administered to 9 hospitalized patients: 3 patients each with one (one died), two, and three doses; one had a booster dose with the Pfizer-BioNTech COVID-19 vaccine. The Sputnik V vaccine was previously administered to 24 hospitalized patients: 5 with one dose, 10 with two doses, and 9 with three doses (one died). The Pfizer-BioNTech vaccine had been previously administered to 35 hospitalized patients: 5 with one dose, 15 with two doses, and 15 with three doses (one had a booster dose with the Sputnik V vaccine). The Sinopharm BIBP COVID-19 vaccine had been previously administered to 227 hospitalized patients: 17 with one dose, 86 with two doses (6 died), and 126 with three doses (7 died and 28 had a booster dose with the Pfizer-BioNTech vaccine (one died) and 1 with the Sputnik vaccine). One patient received three doses of the Moderna COVID-19 vaccine. In total, 48 hospitalized patients died of COVID-19, and 16 of the 48 deceased patients were vaccinated. Specifically, 32 out of 404 unvaccinated patients (7.92%) died, while 16 out of 298 vaccinated patients (5.37%) died. The median age for vaccinated patients who died was 74 (IQR: 66–82), while the median age for unvaccinated patients who died was 76 (IQR: 61–83). The vaccinated COVID-19 patients who died had 100% comorbidities, while unvaccinated COVID-19 patients who died had 93.75% comorbidities. Moreover, 62.5% of deceased vaccinated patients had received a booster dose. In accordance with the previous results related to gross COVID-19 patients, we observed the inflammation biomarkers and liver enzymes related to specific SARS-CoV-2 variants (Figure 4). Vaccines were the most administered to patients with the omicron variant, as the latest observed patients. Compared to females, IL-6 (Mann–Whitney U = 2653, *p* = 0.0241) and CRP (Mann–Whitney U = 7350, *p* = 0.0055) were increased in omicron-variant COVID-19 male patients, while IL-6 also increased in the delta variant (Mann–Whitney U = 3200, *p* = 0.0254, Figure 4A). Fibrinogen (Mann–Whitney U = 7417, *p* = 0.0095) and D-dimer (Mann–Whitney U = 1163, *p* = 0.0235) were increased in the omicron and alpha variants, respectively, of COVID-19 male patients compared to female patients (Figure 4B). Ferritin was significantly more increased in COVID-19 male patients infected by Wuhan-1, delta, and omicron SARS-CoV-2 variants than in female patients (Figure 4C). LDH (Mann–Whitney U = 3697, *p* = 0.0016) and AST (Mann–Whitney U = 3422, *p* = 0.0001) were both increased in the delta variant, and in the Wuhan-1 and omicron variants, in male patients (Figure 4D). In addition, the liver enzymes ALT and GGT were generally significantly more increased in all SARS-CoV-2 variants that infected COVID-19 male patients, as opposed to. female patients, except the alpha variant (Figure 4E,F).

## 4. Discussion

We determined that inflammation (CRP, IL-6, fibrinogen, LDH, ferritin) and coagulation (D-dimer, PT) parameters and liver enzymes (AST, ALT, GGT) were preferentially increased in male COVID-19 patients. IL-6 and the severity of clinical outcome were generally in positive correlation with inflammation (CRP, LDH, ferritin) and coagulation parameters (D-dimer, PT, International Normalized Ratio (INR)). Moreover, inflammatory blood cells and liver enzymes were generally increased in severe clinical outcomes. We showed that creatinine and urea were in strong positive correlation with clinical outcome, while it has been reported that the most important predictors of COVID-19 mortality were age, glomerular filtration, urea, CRP, ferritin, ALT, creatinine, and leukocytes [17,18,19]. Furthermore, IL-6, CRP, D-dimer, and ferritin were significantly higher in severe forms of COVID-19 [20,21]. We showed augmented ferritin and liver enzymes preferentially in COVID-19 male patients, regardless of SARS-CoV-2 variant. We found a very strong correlation between clinical outcome and creatinine, AST, and RDW in female COVID-19 patients as biomarkers for predicting their outcome. The same apply for urea, CK, and ferritin in male COVID-19 patients.

The risks of COVID-19 mortality and hospital admission were for the following comorbidities: liver cirrhosis, neurological conditions, chronic kidney disease, blood cancer, coronary heart disease, and type 2 diabetes after vaccination [22,23]. We observed that comorbidities were more frequent in female COVID-19 patients, while comorbidities were generally in positive correlation with the severity of clinical outcome of COVID-19 patients. Hypertension, diabetes, and obesity were risk factors in most age groups of COVID-19 patients [24]. We also observed hypertension and diabetes as the most frequent comorbidities in COVID-19, while diabetes was 2.5-fold more frequent in female patients. Advanced age, comorbidities, and elevated IL-6 levels were associated with severe COVID-19 [25]. Age was positively associated with the severity of clinical outcome in presented study.

In a previous report, considering immune response, there were no differences in the amount of IgG or IgM antibodies between male and female COVID-19 patients, while female COVID-19 patients had more abundant activated and terminally differentiated T cell populations than male patients [26,27]. We demonstrated initially similar immune response, while temporary higher for IgG in male post-COVID-19 patients after 10 weeks. These variations in immune response demonstrated transient sex difference that stabilize in post-COVID-19 patients. 

The immune response depends on the amount of SARS-CoV-2 at the time of infection, where we showed that the Ct values of RT-qPCR diagnostics were negatively correlated with IL-6 in male COVID-19 patients, indicating that increased IL-6 corresponds to higher viral load. We previously reported that inflammatory cytokines were increased in COVID-19 patients, while IL-6 was also elevated in vaccinated COVID-19 patients [5]. We now showed that IL-6 was positively correlated with clinical outcome regardless of vaccination status, suggesting that anti-inflammatory therapy is important even for vaccinated COVID-19 patients. Patients admitted to the ICU and non-survivors had significantly lower Ct values than those admitted to the ward who recovered from COVID-19, respectively [28]. In conflicting reports, the former showed that Ct values were not associated with age and sex of the COVID-19 patients [28,29], but the latter revealed that male sex and older age showed lower Ct values with higher infectious potential [30]. There was a significant sex difference in Ct values after hospital admission for omicron infections, i.e., lower in women [31]. No significant effect of vaccination on Ct value has been reported in infected persons with the delta variant, nor when controlling for sex [32]. In contrast, Ct values were higher in vaccinated individuals and those with a prior infection [31,33]. 

As a measure of preventing infection and hyperinflammatory response, vaccination was in negative correlation with liver enzymes and LDH in female COVID-19 patients in the present study. People with a breakthrough infection after full vaccination were more likely to be older and female [34]. Infection with alpha, gamma, or delta variants resulted in a higher hospitalization risk, with vaccination reducing that risk [34]. Phylogenetic analysis of more than 2000 SARS-CoV-2 sequences from Serbia showed the dynamic of SARS-CoV-2 genome changes, from March 2020 to January 2023, where the types and positions of the mutations and variants were in accordance with the worldwide reports [16]. We showed that vaccination rescued a patient infected with the B.1.617.2.46.6 sub-sublineage that induced a critical clinical outcome. Vaccinated COVID-19 and post-COVID-19 participants had lower concentrations of inflammatory markers than unvaccinated participants [35]. We presented that vaccination reduced inflammation parameters and the severity of clinical outcome in male COVID-19 patients, and generally reduced the risk of pneumonia. The unvaccinated patients died 47.5% more often than vaccinated COVID-19 patients. Therefore, vaccination reduced, but did not prevent, the mortality of COVID-19 patients.

Limitations of our report are the single-centre study, reducing the number of patients, and the prevalence of male COVID-19 patients, which potentially obscures the difference compared to female COVID-19 patients. A limitation in the interpretation of the results can be attributed to generally small correlation coefficients, but this can be verified by high statistical significance in half of the presented results. Future studies should address the practical application of these biomarkers in the prognosis and clinical outcome of COVID-19. They should include translational research to examine the effects of SARS-CoV-2 in vitro and define its mechanisms of activation of the immune response and inflammatory signaling pathways.

## 5. Conclusions

Inflammation biomarkers were significantly more increased in male COVID-19 patients and generally with the severity of clinical outcome. Comorbidities were more frequent in female COVID-19 patients and commonly with the severity of clinical outcome. The immune response was temporarily higher in male post-COVID-19 patients, while the absence of vaccination was accompanied by increased liver enzymes, inflammation parameters, and the severity of clinical outcome in male COVID-19 patients. Liver enzymes and ferritin were generally increased in male COVID-19 patients regardless of the SARS-CoV-2 variant. We found biomarkers for predicting clinical outcome in COVID-19 patients: creatinine, AST, and RDW in females; and urea, CK, and ferritin in males. Vaccination was associated with a reduced requirement for corticosteroid and anti-inflammatory therapy and contributed to decreased mortality, although it did not entirely eliminate the risk of death in patients with COVID-19. Elevated IL-6 levels were linked with augmented clinical severity regardless of vaccination status, indicating that anti-inflammatory treatment remains important even in vaccinated patients with COVID-19.

## Figures and Tables

**Table 1:** Laboratory parameters of hospitalized COVID-19 male patients in correlation with the severity of clinical outcome and vaccine status. Sp. r—Spearman r; WBC—white blood cell; LYMPH—lymphocytes; MONO—monocytes; #—absolute count; RDW—red cell distribution width; WBC—white blood cell; CK—creatine kinase; CRP—C-reactive protein; PLT—platelets; AST—aspartate aminotransferase, ALT—alanine transaminase, GGT—gamma-glutamyltransferase; INR—International Normalized Ratio, LDH—lactate dehydrogenase; Clin. —clinical. The column “Vaccine” refers to whether the COVID-19 patient was vaccinated or not. The column “N^o^ of vaccine doses” refers to the number of vaccine doses.

Males	Ct Value of PCR			IL-6			Clinical Outcome			Vaccine			N^o^ of Vaccine Doses		
	Sp. r	95% CI	*p*	Sp. r	95% CI	*p*	Sp. r	95% CI	*p*	Sp. r	95% CI	*p*	Sp. r	95% CI	*p*
WBC	0.186	0.048–0.3166	0.0068	0.1365	0.027–0.24	0.0117	0.1343	0.037–0.23	0.0054	0.1283	0.028–0.23	0.0095	0.11	0.017–0.21	0.0182
LYMPH #	0.164	0.0255–0.296	0.0171	−0.19	−0.29–0.082	0.0004	−0.1962	−0.288–0.1	< 0.0001						
MONO#	0.139	0.0005–0.273	0.043	−0.105	−0.21–0.0044	0.0529	−0.1903	−0.28–0.09	< 0.0001	0.1657	0.067–0.26	0.0008	0.179	0.083–0.27	0.0002
PLT	0.251	0.116–0.377	0.0002	−0.1406	−0.25–0.032	0.0094									
IL-6	−0.168	−0.312–0.015	0.0265				0.3963	0.3–0.48	< 0.0001						
UREA				0.1587	0.05–0.264	0.0034	0.2547	0.161–0.34	< 0.0001	0.1371	0.037–0.23	0.0058	0.164	0.067–0.26	0.0007
CREATININE				0.1818	0.074–0.286	0.0008	0.1757	0.079–0.27	0.0003						
INR				0.2349	0.127–0.338	0.0001	0.1926	0.09–0.287	0.0001	−0.141	−0.24–0.04	0.0053	−0.111	−0.21–0.011	0.0252
CK				0.196	0.088–0.3	0.0003	0.2057	0.11–0.298	< 0.0001	−0.1121	−0.21–0.01	0.0245	−0.17	−0.26–0.07	0.0006
D-DIMER				0.1952	0.087–0.298	0.0003	0.1796	0.08–0.273	0.0002						
CRP				0.4152	0.32–0.5	<0.0001	0.3541	0.266–0.44	< 0.0001						
AST				0.2677	0.163–0.37	< 0.0001	0.2442	0.15–0.33	<0.0001	−0.1351	−0.23–0.04	0.0065	−0.18	−0.27–0.08	0.0002
ALT	0.177	0.039–0.308	0.01							−0.1678	−0.26–0.07	0.0007	−0.18	−0.27–0.08	0.0002
GGT							0.1302	0.029–0.23	0.0095	−0.1536	−0.25–0.05	0.0028	−0.14	−0.24–0.04	0.0048
FERRITIN				0.2804	0.167–0.386	< 0.0001	0.2508	0.14–0.356	< 0.0001	−0.1954	−0.31–0.08	0.0008	−0.19	−0.3–0.08	0.0007
FIBRINOGEN				0.1617	0.052–0.27	0.0032	0.1197	0.021–0.22	0.0145						
LDH				0.283	0.179–0.38	< 0.0001	0.3374	0.25–0.42	< 0.0001	−0.2443	−0.34–0.15	<0.0001	−0.28	−0.37–0.190	< 0.0001
Clin. outcome	−0.104	−0.239–0.036	0.133	0.3963	0.294–0.48	< 0.0001				−0.1122	−0.21–0.01	0.0236	−0.10	−0.2–0.002	0.0387
RDW							0.1527	0.06–0.25	0.0016	0.1748	0.08–0.27	0.0004	0.176	0.08–0.27	0.0003
N^o^ of patients	up to 211			up to 340			up to 427			up to 407			up to 427		

**Table 2:** Laboratory parameters of hospitalized COVID-19 female patients in correlation with the severity of clinical outcome and vaccine status. Sp. r—Spearman r; WBC—white blood cell; LYMPH—lymphocytes; MONO—monocytes; #—absolute count; RDW—red cell distribution width; WBC—white blood cell; CK—creatine kinase; CRP—C-reactive protein; PLT—platelets; AST—aspartate aminotransferase, ALT—alanine transaminase, INR—International Normalized Ratio, GGT—gamma-glutamyltransferase; LDH—lactate dehydrogenase; Clin. —clinical. The column “Vaccine” refers to whether the COVID-19 patient was vaccinated or not. The column “N^o^ of vaccine doses” refers the number of vaccine doses.

Female	IL-6			Clinical Outcome			Vaccine			N^o^ of Vaccine Doses		
	Sp. r	95% CI	*p*	Sp. r	95% CI	*p*	Sp. r	95% CI	*p*	Sp. r	95% CI	*p*
WBC	0.180	0.04–0.315	0.011	0.159	0.038–0.27	0.0083						
LYMPH #	−0.16	−0.3–0.017	0.025	−0.180	−0.29–0.06	0.0028	0.144	0.02–0.26	0.0192			
MONO#							0.131	0.008–0.25	0.0324	0.133	0.011–0.25	0.0276
PLT	−0.22	−0.35–0.08	0.002									
IL-6				0.440	0.317–0.55	<0.0001						
UREA	0.145	0.002–0.28	0.041	0.309	0.195–0.42	<0.0001				0.149	0.027–0.26	0.0137
CREATININE	0.194	0.05–0.33	0.006	0.303	0.19–0.41	<0.0001						
INR	0.184	0.041–0.32	0.01	0.184	0.062–0.3	0.0025						
CK	0.175	0.03–0.31	0.015	0.141	0.019–0.26	0.0198						
D-DIMER	0.178	0.04–0.31	0.012	0.290	0.17–0.398	<0.0001						
CRP	0.533	0.42–0.63	<0.0001	0.317	0.203–0.42	<0.0001						
AST	0.369	0.24–0.49	<0.0001	0.266	0.15–0.376	<0.0001	−0.251	−0.36–0.13	<0.0001	−0.24	−0.35–0.12	<0.0001
ALT							−0.196	−0.31–0.07	0.0014	−0.2	−0.32–0.08	0.0008
GGT	0.225	0.08–0.36	0.002	0.155	0.03–0.28	0.0133						
FERRITIN	0.317	0.175–0.45	<0.0001	0.261	0.12–0.39	0.0002						
FIBRINOGEN	0.254	0.115–0.38	0.0003									
LDH	0.375	0.24–0.49	<0.0001	0.382	0.27–0.48	<0.0001	−0.227	−0.34–0.11	0.0002	−0.2	−0.317–0.08	0.0008
Clin. outcome	0.44	0.317–0.549	<0.0001				−0.125	−0.25–0.001	0.0413			
RDW				0.258	0.14–0.37	<0.0001						
N^o^ of patients	up to 199			up to 275			up to 266			up to 275		

**Table 3:** Multivariate logistic regression analysis with clinical outcome as dependent variable. C-reactive protein (CRP), lactate dehydrogenase (LDH), lymphocytes (LYMPH), odds ratios (OR), coefficient β (B), standard error (S.E.), Wald statistic (Wald), significance (*p*-value) (Sig.), confidence interval (C.I.).

	B	S.E.	Wald	df	Sig.	OR	95% C.I. for OR	
							Lower	Upper
All patients								
Age	0.020	0.007	8.086	1	0.004	1.020	1.006	1.034
UREA	0.098	0.028	12.384	1	<0.001	1.103	1.044	1.165
CRP	0.007	0.001	24.175	1	<0.001	1.007	1.004	1.010
LDH	0.005	0.001	31.573	1	<0.001	1.005	1.003	1.006
Vaccine	−0.655	0.218	9.028	1	0.003	0.520	0.339	0.796
Males								
UREA	0.170	0.040	17.993	1	<0.001	1.186	1.096	1.283
CRP	0.008	0.002	19.227	1	<0.001	1.008	1.004	1.012
LDH	0.004	0.001	14.234	1	<0.001	1.004	1.002	1.006
Vaccine	−0.542	0.268	4.082	1	0.043	0.582	0.344	0.984
Females								
Age	0.033	0.011	9.728	1	0.002	1.034	1.012	1.055
LDH	0.008	0.002	23.089	1	<0.001	1.008	1.005	1.012
LYMPH %	−0.064	0.019	11.084	1	0.001	0.938	0.903	0.974

## Data Availability

The raw data supporting the conclusions of this article will be made available by the authors on request.

## Supplementary Materials

The following supporting information can be downloaded at: https://www.mdpi.com/article/10.3390/biomedicines13081995/s1, Supplemental Scheme S1: Flowchart with inclusion and exclusion criteria of COVID-19 patients for enrolment in study; Supplemental Table S1: Sex difference in clinical and laboratory data of COVID-19 patients on hospital admission; Supplemental Table S2: Sex difference in individual comorbidities; Supplemental Table S3: Sex difference in laboratory data of patients with COVID-19, on hospital admission, according to severity of clinical outcome; Supplemental Table S4: Laboratory parameters of hospitalized COVID-19 male and female patients in correlation with the inflammatory cytokine IL-6, severity of clinical outcome and vaccine status; Supplemental Table S5: Co-morbidities of hospitalized COVID-19 patients in correlation with the severity of clinical outcome and sex; Supplemental Table S6: CT Severity Score in vaccinated and unvaccinated patients with COVID-19.
