# Supplementary material for: Prognostic Impact of Vaccination, Comorbidity, and Inflammatory Biomarkers on Clinical Outcome in Hospitalized Patients with COVID-19
Source: Biomedicines. 2025 Aug 16;13(8):1995. doi: 10.3390/biomedicines13081995 (PMC12383355; doi:10.3390/biomedicines13081995)
Supplement: Supplementary file 1 [file biomedicines-13-01995-s001.zip › biomedicines-3713602-supplementary.pdf]

Supplemental scheme S1. Flowchart with inclusion and exclusion criteria of COVID-19 patients for enrolment in study.

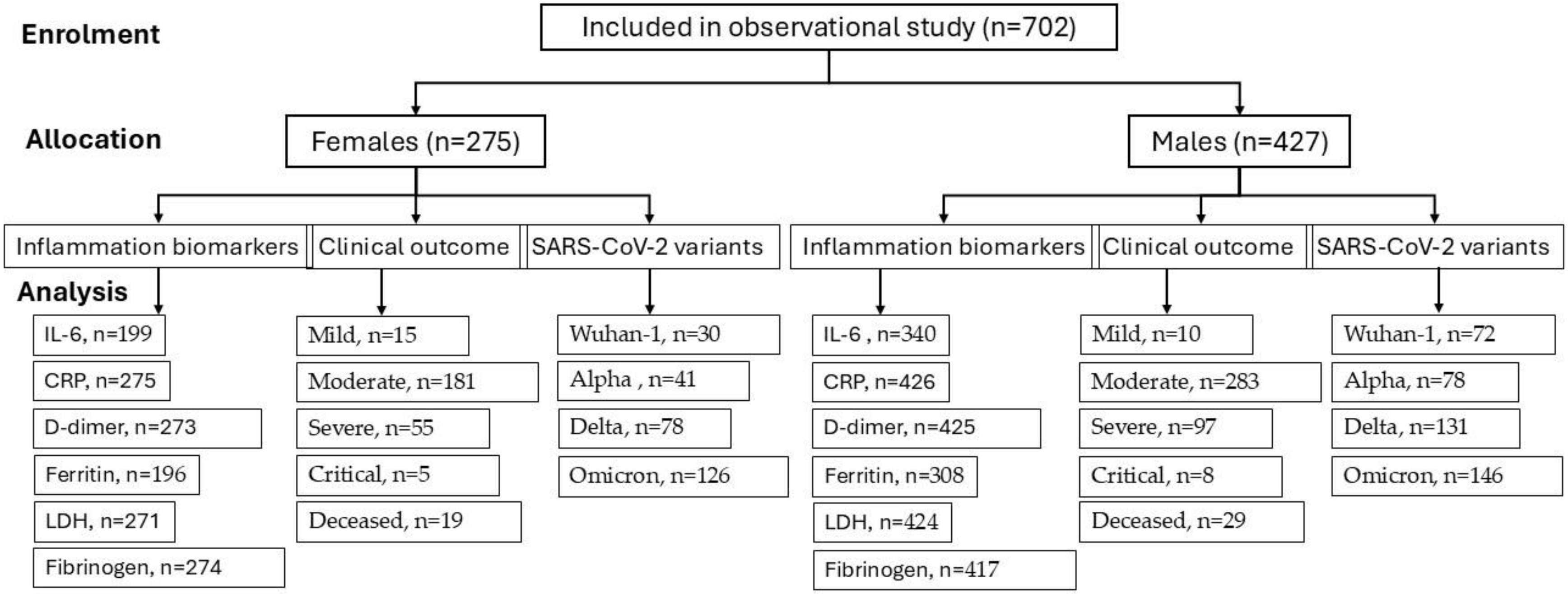

Criteria for COVID-19:	Inclusion	Exclusion
Clinical presentation	mild, moderately and severe	asymptomatic or very mild
pulmonary auscultation	positive	negative
X-ray signs of pneumonia	positive	negative
SpO2	< 90%	> 94%
CRP	> 50 mg/L	< 50 mg/L
Nasopharyngeal swab (PCR, Ag test)	positive	

Supplemental Table S1. Sex difference in clinical and laboratory data of COVID-19 patients on hospital admission.

	Male				Female				M vs. F
	Median	25th percentile	75th percentile	№	Median	25th percentile	75th percentile	№	P value
RBC (10^{12}/L)	4.73	4.35	5.12	427	4.36	4.05	4.72	275	<0.0001
HGB (g/L)	143	133	151	427	129	119	137	275	<0.0001
HCT (L/L)	0.42	0.39	0.45	425	0.382	0.35	0.41	275	<0.0001
NEUT # (10^9L)	4.59	3.02	6.94	427	4.23	2.92	5.93	275	0.06
MCH (pg)	30.1	29	31	427	29.6	28.15	30.8	275	0.0006
MCHC (g/L)	338	331	345	427	333	326.5	340	275	<0.0001
RDW (%)	12.7	12.1	13.4	426	13.05	12.4	14.18	274	<0.0001
MPV (fL)	10	9.5	10.62	424	10	9.5	10.6	273	<0.0001
PLT (10^9L)	189	149	243.5	427	212	162	271	275	<0.0001
PDW (%)	11.1	10.1	12.5	425	11	9.9	12.4	271	0.001
aPTT (sec)	34.6	30.62	37.58	408	32	28.4	34.7	268	<0.0001
PT (%)	79.45	71.2	91.42	62	85.2	76	96.8	44	<0.0001
UREA mmol/L	6.2	4.8	8.3	290	5.6	3.7	7.75	204	<0.0001
Creatinine μmol/L	96	84	114	424	77	63.5	94	275	<0.0001
CK (U/L)	127	73	264.2	425	77	50	144	275	<0.0001
Comorbidities	1	0	1	427	1	1	1	275	0.0006

Bolded values are significantly different between F and M. Weight (1-normal, 2-overweight). RBC-red blood cell; HGB-hemoglobin; HCT-hematocrit; MCH- mean corpuscular hemoglobin; MCHC- mean corpuscular hemoglobin concentration; RDW-red cell distribution width; NEUT-neutrophils; MPV-mean platelet volume; PLT-platelets; PDW-platelet distribution width; INR-international normalized ratio; aPTT-activated partial thromboplastin time; PT-prothrombin time; CK-creatine kinase.

Supplemental Table S2. Sex difference in individual comorbidities.

**Comorbidities**	**Female (%)**	**Male (%)**	**Comorbidities**	**Female (%)**	**Male (%)**
total	79.64	67.92	gastrointestinal	8.00	7.03
cardiovascular	57.45	51.52	genitourinary system	7.64	10.07
metabolic	3.27	3.28	dermatology	3.64	3.75
thromboembolic	1.82	1.64	neurological	7.27	7.26
diabetes mellitus	21.09	16.63	psychiatric	5.45	3.51
endocrinological	16.36	1.87	ophthalmological	0.36	1.41
malignancies	13.82	9.84	sepsis	0.73	1.17
respiratory	11.27	8.90	autoimmune	8.36	2.81
blood and blood-forming organs	6.91	6.09	musculoskeletal system and connective tissue	4.00	2.34

Supplemental Table S3. Sex difference in laboratory data of patients with COVID-19, on hospital admission, according to severity of clinical outcome.

	percentile	Female		Male		Female p value	Male p value	Female vs. male p value	
Clinical outcome category		1-2	3-5	1-2	3-5	1-2 vs. 3-5		1-2	3-5
PT (%)	Median	86.3	82.5	82	72.5		0.0004	0.0116	
	25th	78.1	69.4	74	67.7				
	75th	96.8	92.6	92.6	86.7				
	n	147	57	205	85				
UREA mmol/L	Median	5.15	6.9	6	7.2	<0.0001	<0.0001	<0.0001	
	25th	3.6	4.8	4.7	5.3				
	75th	6.8	10.4	7.4	10.5				
	n	196	79	290	134				
Creatinine µmol/L	Median	74	88	94	102.5	<0.0001	0.0003	<0.0001	0.0019
	25th	61.8	71.5	81	88				
	75th	88	121	110	121.5				
	n	196	79	291	134				
CK (U/L)	Median	73	109	118.5	171	0.0196	0.0003	<0.0001	0.0005
	25th	48.8	56	64.2	81.8				
	75th	124.2	175	224.2	353.2				
	n	192	79	290	132				
Vaccine	Median	0	0	0	0		0.0244		
Comorbidities	Median	1	1	1	1	0.0204		0.0191	0.0092

(1-2) - mild clinical outcome, (3-5) - severe clinical outcome. PT-prothrombin time; CK-creatine kinase.

Supplemental Table S4. Laboratory parameters of hospitalized COVID-19 male and female patients in correlation with the inflammatory cytokine IL-6, severity of clinical outcome and vaccine status.

Males	IL-6				Clinical outcome				Vaccine				№ of vaccine doses			
	Males		Females		Males		Females		Males		Females		Males		Females	
	p	q	p	q	p	q	p	q	p	q	p	q	p	q	p	q
WBC	0.0117	0.0055	0.0108	0.0068	0.0054	0.0023	0.0083	0.0058	0.0095	0.0145	0.0697	0.153	0.0182	0.0278	0.1429	0.2876
LYMPH #	0.0004	0.0003	0.0245	0.0129	0.0001	0.0001	0.0028	0.0021	0.9074	0.5646	0.0192	0.0927	0.9938	0.6184	0.1203	0.2641
MONO#	0.0529	0.0222	0.2624	0.1078	0.0001	0.0001	0.507	0.2129	0.0008	0.0022	0.0324	0.1118	0.0002	0.0007	0.0276	0.0952
PLT	0.0094	0.0049	0.0017	0.0016	0.1114	0.0349	0.0778	0.043	0.4226	0.341	0.4372	0.5395	0.8232	0.5532	0.4505	0.5726
IL-6					0.0001	0.0001	0.0001	0.0001	0.8547	0.5646	0.2248	0.3193	0.6447	0.4709	0.1974	0.3178
UREA	0.0034	0.0019	0.0411	0.0204	0.0001	0.0001	0.0001	0.0001	0.0058	0.0108	0.059	0.153	0.0007	0.0013	0.0137	0.066
CREATININE	0.0008	0.0005	0.006	0.0047	0.0001	0.0001	0.0001	0.0001	0.4365	0.341	0.7672	0.772	0.962	0.6184	0.4285	0.5726
INR	0.0001	0.0001	0.01	0.0068	0.0003	0.0001	0.0025	0.002	0.0053	0.0108	0.1612	0.2995	0.0252	0.0353	0.5017	0.577
CK	0.0003	0.0002	0.0147	0.0082	0.0001	0.0001	0.0198	0.0122	0.0245	0.0317	0.3898	0.523	0.0006	0.0013	0.5962	0.626
D-DIMER	0.0003	0.0002	0.012	0.0071	0.0002	0.0001	0.0001	0.0001	0.2464	0.2435	0.9812	0.8776	0.1361	0.1488	0.5956	0.626
CRP	0.0001	0.0001	0.0001	0.0002	0.0001	0.0001	0.0001	0.0001	0.3497	0.3092	0.5564	0.6108	0.3999	0.3255	0.7463	0.7209
AST	0.0001	0.0001	0.0001	0.0002	0.0001	0.0001	0.0001	0.0001	0.0065	0.0109	0.0001	0.0012	0.0002	0.0007	0.0001	0.0012
ALT	0.3305	0.1207	0.9715	0.34	0.1115	0.0349	0.8869	0.3449	0.0007	0.0022	0.0014	0.0085	0.0002	0.0007	0.0008	0.0048
GGT	0.1797	0.0719	0.0022	0.0019	0.0095	0.0039	0.0133	0.0087	0.0028	0.0067	0.0655	0.153	0.0048	0.0081	0.0501	0.1512
FERRITIN	0.0001	0.0001	0.0001	0.0002	0.0001	0.0001	0.0002	0.0002	0.0008	0.0022	0.1904	0.3065	0.0007	0.0013	0.1667	0.2876
FIBRINOGEN	0.0032	0.0019	0.0003	0.0005	0.0145	0.0056	0.2967	0.1355	0.3052	0.2849	0.1529	0.2995	0.218	0.1928	0.0604	0.1621
LDH	0.0001	0.0001	0.0001	0.0002	0.0001	0.0001	0.0001	0.0001	0.0001	0.0008	0.0002	0.0016	0.0001	0.0007	0.0008	0.0048
Clin. outcome	0.0001	0.0001	0.0001	0.0002					0.0236	0.0317	0.0413	0.1247	0.0387	0.05	0.1606	0.2876
RDW	0.8201	0.265	0.0647	0.0306	0.0016	0.0007	0.0001	0.0001	0.0004	0.0022	0.5988	0.6287	0.0003	0.0008	0.2284	0.3447

p - value of nonparametric Spearman corelation; q - the minimum False Discovery Rate (q=5%) at which a particular test can be called significant (the adjusted p-value).

Supplemental Table S5. Co-morbidities of hospitalized COVID-19 patients in correlation with the severity of clinical outcome and sex.

Diseases	Sex	Clinical outcome			
		Sp. r	95% CI	p	№
All co-morbidities	Total	0.111	0.035 - 0.186	0.0032	510
	Male	**0.116**	**0.006 - 0.22**	**0.033**	**291**
	Female	0.193	0.07 - 0.31	0.0013	219
Cardiovascular	Total	0.154	0.078 - 0.23	<0.0001	379
	Female	0.286	0.17 - 0.39	<0.0001	159
Pulmonary	Total	0.186	0.11 - 0.26	<0.0001	69
	Male	0.156	0.06 - 0.25	0.0012	38
	Female	0.232	0.11 - 0.34	0.0001	31
Psychiatric	Total	0.164	0.089 - 0.24	<0.0001	30
	Male	0.107	0.01 - 0.2	0.0265	15
	Female	0.239	0.12 - 0.35	<0.0001	15
Blood and blood-forming organs	Total	0.08	0.004 - 0.15	0.0347	45
	Total	**0.085**	**-0.002 - 0.17**	**0.049**	**45**
Urinary-genital system	Female	0.145	0.023 - 0.26	0.0165	21
Diabetes mellitus	Female	**0.156**	**0.012 - 0.29**	**0.03**	**58**

Bolded values are vs. IL-6, Spearman r - Sp. r.

Supplemental Table S6. CT Severity Score in vaccinated and unvaccinated patients with COVID-19.

COVID-19 patients	Unvaccinated			Vaccinated		
CT severity score	n	Clinical outcome ranking				n
		Mean	Median	Mean	Median	
2				2	2	1
3	2	2	2	2	2	1
4	1	2	2	3	3	1
5	6	2.17	2	2	2	3
6	3	2	2	2	2	3
7	6	2.33	2	3	2	3
8	5	2.6	3	3.2	3	5
9	9	2.22	2	2	2	3
10	7	2.29	2	2.44	2	9
11	8	2.63	2	2.3	2	10
12	20	2.30	2	2.13	2	8
13	7	2.57	3	2.2	2	10
14	12	2.25	2	3	2	7
15	14	2.71	3	2.6	3	10
16	6	2.83	2.5	2.67	3	3
17	8	2.75	3	2.6	3	5
18	3	3.33	3	3	3	1
19	1	3	3			
20	3	3.33	3			
21	2	3.5	3.5			
22	1	3	3			

CT – computed tomography, clinical outcome ranking is 1-5. CT Severity Score ≥8 is indication of clinically relevant COVID-19 pneumonia.
